# Evaluating the impact of vitreomacular adhesion on anti-VEGF therapy for retinal vein occlusion using machine learning

**DOI:** 10.1038/s41598-017-02971-y

**Published:** 2017-06-07

**Authors:** Sebastian M. Waldstein, Alessio Montuoro, Dominika Podkowinski, Ana-Maria Philip, Bianca S. Gerendas, Hrvoje Bogunovic, Ursula Schmidt-Erfurth

**Affiliations:** Christian Doppler Laboratory for Ophthalmic Image Analysis Vienna Reading Center, Department of Ophthalmology, Medical University of Vienna Spitalgasse 23, AT-1090 Vienna, Austria

## Abstract

Vitreomacular adhesion (VMA) represents a prognostic biomarker in the management of exudative macular disease using anti-vascular endothelial growth factor (VEGF) agents. However, manual evaluation of VMA in 3D optical coherence tomography (OCT) is laborious and data on its impact on therapy of retinal vein occlusion (RVO) are limited. The aim of this study was to (1) develop a fully automated segmentation algorithm for the posterior vitreous boundary and (2) to study the effect of VMA on anti-VEGF therapy for RVO. A combined machine learning/graph cut segmentation algorithm for the posterior vitreous boundary was designed and evaluated. 391 patients with central/branch RVO under standardized ranibizumab treatment for 6/12 months were included in a systematic post-hoc analysis. VMA (70%) was automatically differentiated from non-VMA (30%) using the developed method combined with unsupervised clustering. In this proof-of-principle study, eyes with VMA showed larger BCVA gains than non-VMA eyes (BRVO: 15 ± 12 vs. 11 ± 11 letters, p = 0.02; CRVO: 18 ± 14 vs. 9 ± 13 letters, p < 0.01) and received a similar number of retreatments. However, this association diminished after adjustment for baseline BCVA, also when using more fine-grained VMA classes. Our study illustrates that machine learning represents a promising path to assess imaging biomarkers in OCT.

## Introduction

Retinal vein occlusion (RVO) is a frequent cause of visual disability in the working-age population^1^. Anti-vascular endothelial growth factor (anti-VEGF) therapy provides an effective treatment option for macular oedema secondary to central and branch RVO in a wide range of patients, including those with longer disease duration and macular ischemia^2–7^. Because the individual functional response to therapy and the number of required treatments over time is highly variable, the establishment of prognostic factors for anti-VEGF therapy represents an active field of research^8, 9^.

In addition to the morphologic status of the neurosensory retina, the configuration of the vitreous and vitreomacular interface has attracted scientific interest as a prognostic factor in anti-VEGF therapy in the recent years^10^. Prior studies observed visually asymptomatic vitreomacular adhesion (VMA) in about 40% of patients with macular oedema secondary to RVO^11^. Studies in neovascular AMD suggest excellent functional response to anti-VEGF therapy in eyes with VMA, if treatment is administered frequently and aggressively^12–14^. A positive impact of VMA on visual acuity outcomes in anti-VEGF therapy has also been reported in diabetic macular oedema and in anti-VEGF therapy for RVO^11, 15, 16^.

The advent of spectral-domain optical coherence tomography (SD-OCT) with its fast raster scanning capabilities, in combination with rising patient numbers in ophthalmology clinics, has led to a substantial increase in OCT images for the clinician to assess. Thus, researchers have started to question the feasibility of a manual review of OCT images in clinical practice, and suggest automated computational analysis of SD-OCT data to aid clinical management and decision making^17^.

In this paper, we use fully automated computational segmentation and classification of SD-OCT scans to evaluate VMA in patients receiving ranibizumab for macular oedema secondary to branch and central RVO in two multicentre prospective clinical trials, and compare visual acuity outcomes between patients with and without VMA.

## Patients and Methods

This study is a post-hoc analysis of a comprehensive clinical trial image library at the Vienna Reading Center (VRC), Vienna, Austria. For central RVO, we included SD-OCT data of patients enrolled in CRYSTAL, a phase IIIb, multicentre trial assessing the efficacy and safety of an individualized visual acuity (VA) dosing regimen of ranibizumab 0.5 mg driven by stabilization criteria^6^. For branch RVO, we included SD-OCT data of patients enrolled in BRIGHTER, a phase IIIb, multicentre study assessing the efficacy and safety of an individualized stabilization criteria–driven pro re nata (PRN) dosing regimen of ranibizumab 0.5 mg alone or in combination with laser versus laser photocoagulation^7^. Patients randomized to the laser only arm of BRIGHTER were excluded from the current analysis because of previously demonstrated inferior VA outcomes^7^. Our study was conducted in compliance with the Declaration of Helsinki. Approval was obtained by the institutional review board or ethics committee at each site participating in the multicentre trial. Furthermore, approval for the present post-hoc analysis was obtained by the Ethics Committee at the Medical University of Vienna. All participants provided written informed consent before inclusion into the CRYSTAL and BRIGHTER trials. The multicentre trials are registered with clinicaltrials.gov (identifiers NCT01599650 and NCT01535261).

### Inclusion and exclusion criteria

Detailed inclusion and exclusion criteria of the CRYSTAL and BRIGHTER studies have been reported previously^6, 7^. In brief, patients with macular oedema secondary to branch or central RVO and a visual acuity between 20/40 and 20/400 (Snellen equivalent) were included. Key exclusion criteria were the use of intraocular anti-VEGF treatment ≤3 months, intraocular corticosteroid use ≤3 months, and macular laser photocoagulation ≤4 months before the screening visit. Because the VMA segmentation algorithm developed for this work (see below) is currently only available for Heidelberg Spectralis OCT images, only data of patients scanned with this particular OCT machine were included into this post-hoc analysis.

### Treatment schedule; functional and structural assessments

All patients analysed in this study received intravitreal 0.5 mg ranibizumab monthly until stable VA was observed for 3 consecutive monthly visits (implying a minimum of 3 consecutive treatments per protocol). The investigators then monitored the patients for VA and disease activity on a monthly basis. Monthly ranibizumab injections were reinitiated if monitoring indicated a loss of VA resulting from disease activity, and were continued until VA stabilization. In BRIGHTER, patients randomized to the ranibizumab with laser arm received macular laser based on investigator discretion as reported previously^7^.

Best-corrected visual acuity (BCVA) was measured by certified, masked examiners at each monthly visit using early treatment diabetic retinopathy study (ETDRS) charts. SD-OCT was acquired at each visit by certified, masked operators. Using Heidelberg Spectralis OCT instruments, a volume scanning pattern of 512 A-scans × 49 B-scans covering 20° × 20° centred on the fovea was acquired. Eye tracking and automated real-time averaging were activated at 29 frames. Raw data was exported and uploaded to the VRC database.

### Automated segmentation of the posterior vitreous boundary

**Figure 1:**
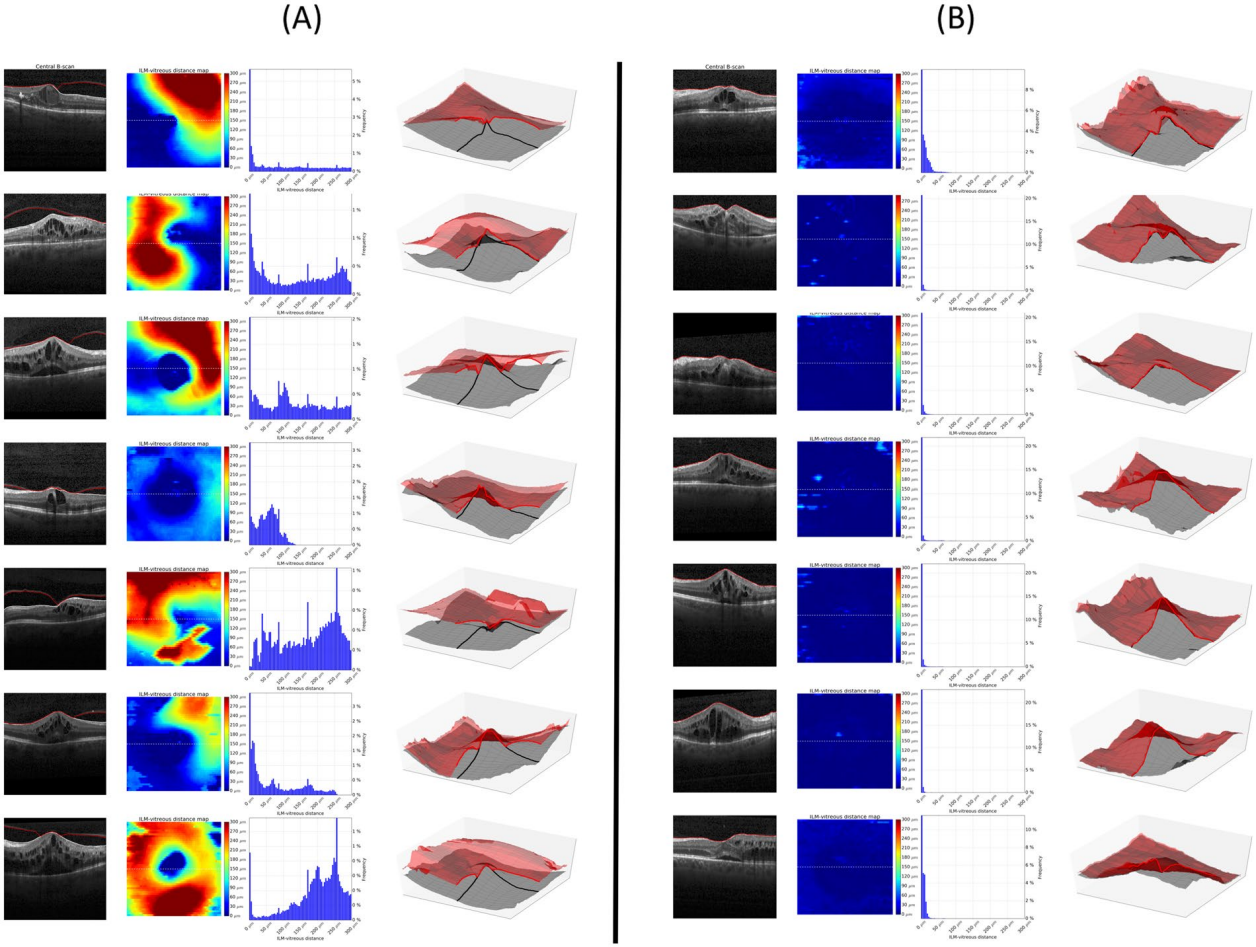
Fully automated segmentation of the posterior vitreous boundary in 3D optical coherence tomography. Panel (A) shows example patients with vitreomacular adhesion, Panel (B) illustrates eyes without vitreomacular adhesion. Far left: Central B-scan showing the segmentation obtained by the automated algorithm (red line = posterior vitreous boundary). Left: *En-face* map of the distance between the internal limiting membrane and the posterior vitreous boundary for the 6 × 6 mm optical coherence tomography volume. Right: Histogram of distances shown in the *en-face* map used as an input feature for unsupervised clustering. Far right: 3D visualization of the internal limiting membrane and the posterior vitreous boundary. The bold line in the 3D visualization corresponds to a 90° wedge being cut out of the volume; note that the line to the right of the image corresponds to the right half of the provided B-scan, while the part pointing towards the observer is perpendicular to the B-scan slice shown on the left.

A custom designed, fully automated segmentation algorithm was used to delineate the posterior vitreous boundary in the SD-OCT volume scans. The software method is described in detail in the section “Description of segmentation algorithm”. Preliminary versions of the method have been reported previously^18, 19^, as well as an extension to retinal fluid and layer segmentation^20^. In brief, a machine learning algorithm was trained to classify voxels belonging to the posterior vitreous boundary in the OCT volumes. The posterior vitreous interface was regarded as a continuous surface and a 3D graph cut segmentation algorithm was used to fit an optimal surface through the voxels classified as posterior vitreous boundary with highest probability. The segmentation algorithm also delineates the internal limiting membrane (ILM). The automated segmentation method was applied on all baseline SD-OCT volume scans to delineate the posterior vitreous boundary and the ILM. The distance between the two segmentation surfaces was computed at each A-scan and presented as ILM-vitreous distance map (Fig. 1).

### Automated classification of vitreomacular adhesion

**Figure 2:**
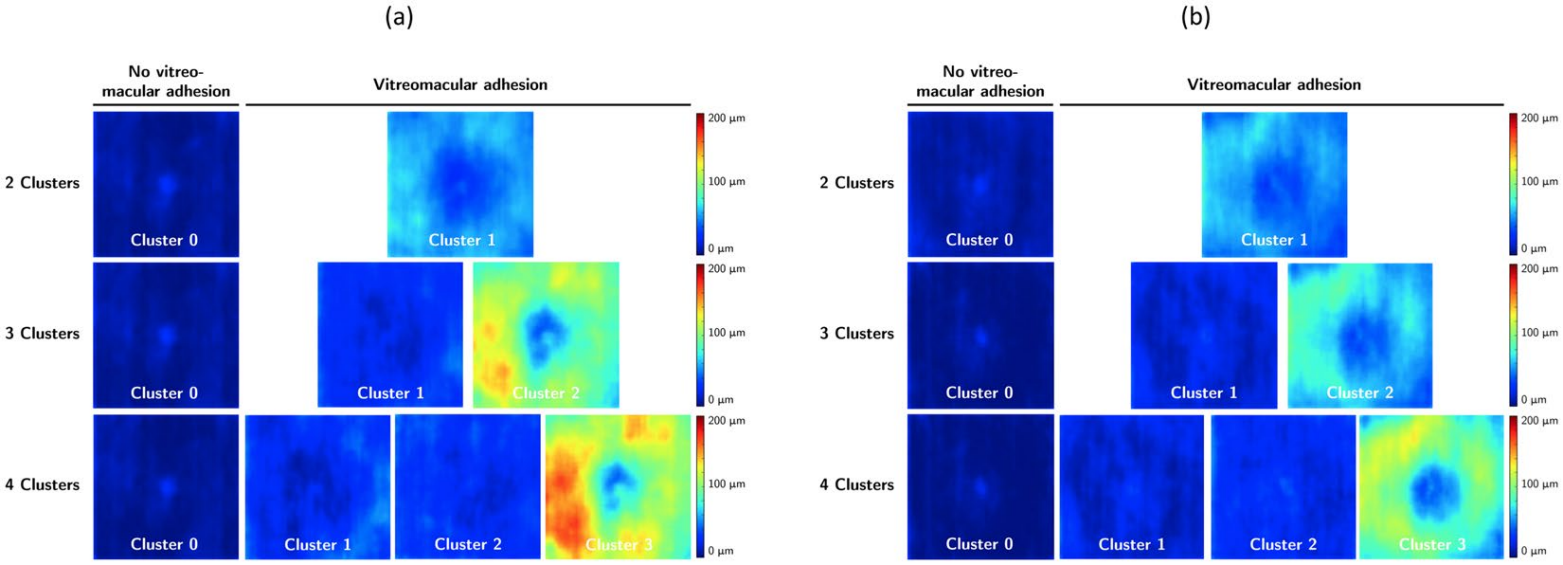
Unsupervised clustering of vitreomacular interface configurations. Panel (a) shows clusters for branch retinal vein occlusion patients; Panel (b) for central retinal vein occlusion. The Cluster 0 contains eyes without vitreomacular adhesion, including both completely attached vitreous and complete posterior vitreous detachment. Clusters 1–3 contain eyes with vitreomacular adhesion. If more clusters are chosen, the unsupervised algorithm differentiates between shallow and steep vitreomacular adhesion in separate classes.

To automatically classify the ILM-vitreous distance maps of each patient into VMA and non-VMA, the histogram of the ILM-vitreous distances at each A-scan of the volume was computed. The resulting histograms were automatically clustered by unsupervised machine learning using the *k*-means algorithm^21^. To evaluate the robustness of the clustering, the number of clusters was systematically varied from 2 to 4. The clusters were interpreted clinically by expert evaluation of the ILM-vitreous distance map representing the cluster centre (Fig. 2).

### Statistical analysis

This is an exploratory post-hoc analysis of a clinical trial database. Hence, no formal hypotheses or adjustments for multiple comparisons were made, and *p*-values are to be viewed as hypothesis-generating. The main outcome measure of this study was the mean change in BCVA at the primary endpoint (Month 6 for BRIGHTER, Month 12 for CRYSTAL). Additional outcome measures were the change in reading-centre determined central retinal thickness (CRT) as well as the number of administered ranibizumab injections at the primary endpoint. The means of the outcome variables were compared between eyes with and without VMA using analysis of variance (ANOVA). Moreover, linear regression analysis was performed to assess the predictive impact of VMA on BCVA change, including the BCVA at baseline as an independent co-variate. The linear regression analysis was performed separately for 2 clusters (VMA presence vs. VMA absence), as well as 3 and 4 clusters (VMA absence vs. different degrees of VMA). The formal significance level was set at *p* = 0.05.

## Results

The main outcomes and patient characteristics of CRYSTAL and BRIGHTER have been published previously^6, 7^. All 812 patients randomized in CRYSTAL (n = 357) and BRIGHTER (n = 455) were screened for the current analysis. Patients receiving macular laser photocoagulation only in BRIGHTER (n = 97) were excluded. Furthermore, patients imaged by OCT devices other than Spectralis OCT were excluded, resulting in 391 patients for analysis (198 in CRYSTAL; 193 in BRIGHTER).

### Automated segmentation and classification of vitreomacular adhesion

Automated segmentation of the posterior vitreous boundary and generation of ILM-vitreous distance maps was successful in all patients. Representative segmentation results and ILM-vitreous distance maps of study eyes are provided in Fig. 1. Unsupervised clustering of the ILM-vitreous distance histograms revealed clinically distinctive classes of vitreomacular interface configurations (Fig. 2). According to the number of selected clusters (2–4), the *k*-means algorithm grouped non-VMA eyes; VMA with shallow vitreomacular separation and VMA with steeper adhesions into separate classes (Fig. 2), which was verified by clinical experts (SMW, BSG) after reviewing the cases. The mean average distances between the ILM and the posterior vitreous boundary for each cluster are provided in Tables 1 and 2.

**Table 1:** Characteristics of vitreomacular interface clusters in branch retinal vein occlusion (BRVO).

	Cluster 0 (Non-VMA)		Cluster 1 (VMA)		*p*
N=	64		129		
Baseline BCVA (mean +− SD)	60.42 +− 12.07		57.09 +− 13.23		0.0941
BCVA change at month 6 (mean +− SD)	11.07 +− 10.75		15.48 +− 11.56		0.0152
Baseline CRT (mean +− SD)	358.21 +− 98.36		555.69 +− 182.82		0.0001
CRT change at month 6 (mean +− SD)	−79.80 +− 75.92		−218.06 +− 197.74		0.0140
Baseline ILM-VIT distance (mean +− SD)	1.69 +− 1.85		13.88 +− 13.58		<0.0001
Number of ranibizumab injections (mean +− SD)	4.72 +− 1.14		4.94 +− 0.90		0.1801
	Cluster 0 (Non-VMA)	Cluster 1 (VMA)	Cluster 2 (VMA)		*p*
N=	62	81	50		
Baseline BCVA (mean +− SD)	60.11 +− 12.14	57.92 +− 13.52	56.28 +− 12.67		0.2916
BCVA change at month 6 (mean +− SD)	10.93 +− 10.80	17.33 +− 10.20	12.38 +− 12.83		0.0031
Baseline CRT (mean +− SD)	362.78 +− 99.07	524.62 +− 135.32	594.50 +− 252.86		0.0008
CRT change at month 6 (mean +− SD)	−85.79 +− 75.09	−198.95 +− 153.10	−234.36 +− 262.61		0.0844
Baseline ILM-VIT distance (mean +− SD)	1.67 +− 1.86	6.67 +− 4.71	25.09 +− 15.34		<0.0001
Number of ranibizumab injections (mean +− SD)	4.67 +− 1.13	4.96 +− 0.89	4.97 +− 0.92		0.2183
	Cluster 0 (Non-VMA)	Cluster 1 (VMA)	Cluster 2 (VMA)	Cluster 3 (VMA)	*p*
N=	58	46	51	38	
Baseline BCVA (mean +− SD)	60.05 +− 12.00	56.87 +− 13.39	59.65 +− 13.40	55.03 +− 12.41	0.2076
BCVA change at month 6 (mean +− SD)	10.98 +− 11.16	16.07 +− 11.36	16.57 +− 8.68	12.69 +− 13.88	0.0473
Baseline CRT (mean +− SD)	355.88 +− 97.65	488.11 +− 106.48	667.73 +− 252.29	509.25 +− 118.28	0.0001
CRT change at month 6 (mean +− SD)	−86.69 +− 110.60	−181.67 +− 149.39	−391.70 +− 278.24	−186.38 +− 164.69	0.0042
Baseline ILM-VIT distance (mean +− SD)	1.53 +− 1.78	6.94 +− 5.89	7.12 +− 3.23	29.67 +− 14.81	<0.0001
Number of ranibizumab injections (mean +− SD)	4.69 +− 1.12	5.05 +− 0.92	4.80 +− 0.92	5.00 +− 0.89	0.3118

BCVA = best-corrected visual acuity; SD = standard deviation; CRT = central retinal thickness; ILM = internal limiting membrane; VIT = posterior vitreous boundary.

**Table 2:** Characteristics of vitreomacular interface clusters in central retinal vein occlusion (CRVO).

	Cluster 0 (non-VMA)			Cluster 1 (VMA)	*p*
N=	82			116	
Baseline BCVA (mean +− SD)	58.35 +− 12.61			50.85 +− 15.52	0.0004
BCVA change at month 12 (mean +− SD)	9.40 +− 12.99			17.65 +− 13.61	0.0004
Baseline CRT (mean +− SD)	630.50 +− 78.50			707.36 +− 300.53	0.7471
CRT change at month 12 (mean +− SD)	−423.50 +− 125.50			−468.40 +− 353.92	0.8747
Baseline ILM-VIT distance (mean +− SD)	2.38 +− 2.47			13.71 +− 12.41	<0.0001
Number of ranibizumab injections (mean +− SD)	8.08 +− 2.74			8.32 +− 2.68	0.6470
	Cluster 0 (non-VMA)	Cluster 1 (VMA)		Cluster 2 (VMA)	*p*
N=	44	68		86	
Baseline BCVA (mean +− SD)	61.00 +− 11.57	54.90 +− 14.23		49.64 +− 15.32	0.0001
BCVA change at month 12 (mean +− SD)	8.41 +− 13.87	13.00 +− 13.65		17.85 +− 13.08	0.0059
Baseline CRT (mean +− SD)	630.50 +− 78.50	485.00 +− 0.00		729.60 +− 306.45	0.7208
CRT change at month 12 (mean +− SD)	−423.50 +− 125.50	−212.00 +− 0.00		−496.89 +− 362.02	0.7593
Baseline ILM-VIT distance (mean +− SD)	1.12 +− 0.75	4.57 +− 3.50		16.58 +− 13.05	<0.0001
Number of ranibizumab injections (mean +− SD)	8.00 +− 2.95	7.78 +− 2.55		8.64 +− 2.61	0.3026
	Cluster 0 (non-VMA)	Cluster 1 (VMA)	Cluster 2 (VMA)	Cluster 3 (VMA)	*p*
N=	40	57	48	53	
Baseline BCVA (mean +− SD)	61.56 +− 11.52	55.54 +− 13.26	49.67 +− 15.74	50.45 +− 15.35	0.0004
BCVA change at month 12 (mean +− SD)	8.97 +− 13.61	11.37 +− 12.89	20.13 +− 17.32	16.27 +− 9.71	0.0058
Baseline CRT (mean +− SD)	630.50 +− 78.50	485.00 +− 0.00	709.50 +− 367.10	743.00 +− 257.35	0.8855
CRT change at month 12 (mean +− SD)	−423.50 +− 125.50	−212.00 +− 0.00	−612.00 +− 441.35	−439.33 +− 298.76	0.8143
Baseline ILM-VIT distance (mean +− SD)	1.07 +− 0.73	4.35 +− 3.74	6.24 +− 3.73	22.56 +− 13.15	<0.0001
Number of ranibizumab injections (mean +− SD)	7.89 +− 2.95	7.77 +− 2.68	8.21 +− 2.94	8.94 +− 2.09	0.3290

BCVA = best-corrected visual acuity; SD = standard deviation; CRT = central retinal thickness; ILM = internal limiting membrane; VIT = posterior vitreous boundary.

### Visual acuity outcomes in VMA versus non-VMA eyes

Mean baseline BCVA and BCVA change from baseline to the primary endpoint in the different VMA classes are provided for CRYSTAL and BRIGHTER in Tables 1 and 2. In CRVO, eyes without VMA showed higher mean baseline BCVA than eyes with VMA, while baseline BCVA was similar in eyes with and without VMA in BRVO. In both CRVO and BRVO, eyes showing VMA achieved better BCVA gains than eyes without VMA. Eyes showing no VMA (Cluster 0) demonstrated overall poorest BCVA gains.

Linear regression models were fitted to evaluate the interaction between baseline BCVA, VMA presence and BCVA change to the primary endpoint. In BRVO (using 2 clusters), the model fit for BCVA change was R = 0.30, *p* = 0.03. BCVA at baseline was a significant predictor (*p* = 0.03) whereas VMA presence was not (*p* = 0.28). For 3 clusters, the model fit was R = 0.28, *p* = 0.05; *p* = 0.01 for baseline BCVA and *p* = 0.74 for VMA class. For 4 clusters, the model fit was R = 0.35, *p* < 0.01; *p* < 0.01 for baseline BCVA and *p* = 0.05 for VMA class. Similarly, in CRVO, for 2 clusters, the model fit was R = 0.36, *p* = 0.01, with BCVA at baseline being a significant predictor (*p* = 0.02) as opposed to VMA presence (*p* = 0.10). Using 3 clusters, the model fit was R = 0.30, *p* = 0.05; *p* = 0.02 for baseline BCVA and *p* = 0.99 for VMA class. For 4 clusters, the model fit was R = 0.32, *p* = 0.04; *p* = 0.02 for baseline BCVA and *p* = 0.42 for VMA class.

### Anatomical outcomes in VMA versus non-VMA eyes

Mean baseline CRT and CRT change at the primary endpoint are provided in Tables 1 and 2. Baseline CRT did not differ among eyes with or without VMA in CRVO, while eyes without VMA showed significantly lower baseline CRT compared to eyes with VMA in BRVO. Correspondingly, eyes with VMA demonstrated significantly larger CRT reductions at the primary endpoint in BRVO. The mean number of ranibizumab injections received up to the primary endpoint did not differ significantly between the vitreomacular interface clusters (Tables 1 and 2).

## Discussion

In this study, we used fully automated computational analysis based on machine learning to characterize the condition of the vitreomacular interface in a large dataset of SD-OCT images. The proposed method demonstrated that fully automated characterization of retinal morphology is feasible in a large-scale randomized clinical trial setting, where manual analysis of SD-OCT images may not be practicable. Our results suggest that patients with VMA may show larger functional gains with individualized anti-VEGF therapy in macular oedema secondary to central and branch RVO, although this effect was not visible any longer when the analysis was adjusted for baseline BCVA. In the era of personalized medicine and the big-data situation entailed by modern OCT imaging, automated analysis of ophthalmic imaging is a promising step with the potential to transform clinical care in the future^22^.

The role of the vitreous in anti-VEGF therapy for exudative macular disease has attracted scientific interest in the recent years. While ample data exists in the setting of neovascular age-related macular degeneration, scientific studies are scarce in macular oedema of other origins. In anti-VEGF therapy of RVO, which was investigated here, Terao *et al*. reported superior functional and anatomical response to bevacizumab in eyes with VMA^11^. However, their study included only branch RVO and was limited in patient number. On the other hand, a recent study by Singh *et al*. reported no influence of VMA on outcomes of intravitreal therapy for RVO^16^. Our results show a positive impact of VMA on outcomes of anti-VEGF therapy (albeit not confirmed when adjusting for baseline BCVA) in a much larger number of patients, including those with central RVO, using data of a prospective randomized controlled trial. Other studies have investigated the role of VMA in anti-VEGF therapy of diabetic macular oedema and of macular oedema in uveitis, with similar results^15, 23^.

The potential reasons for the interaction between VMA and the response to intravitreal therapy are currently poorly understood. Since the vitreous acts as a reservoir for anti-VEGF agents injected into the eye, pharmacokinetic mechanisms influencing drug half-life or diffusion in senile vitreous liquefaction or PVD may be responsible for some of the observed phenomena^12^. Although the distribution of drug molecules inside the vitreous cavity is governed by passive diffusion, the interaction between the vitreous gel and charged large molecules, as well as vitreous fluid currents may play a role in drug distribution and clearance within the eye^24, 25^. Potentially, egress of VEGF molecules from the retina is altered by the presence of aqueous instead of vitreous at the vitreomacular interface^26^. Laboratory studies have demonstrated a faster clearance of anti-VEGF agents from vitrectomised eyes, which would suggest a shorter half-life of ranibizumab also in eyes with complete PVD and/or senile vitreous liquefaction^27^. A relatively prolonged clearance of anti-VEGF molecules from eyes with VMA could explain the beneficial functional and anatomical outcomes in the presence of VMA in this and other studies. However, in neovascular age-related macular degeneration, the presence of a posterior vitreous detachment (PVD) also seemed to be associated with reduced needs for retreatment, which clearly contradicts this hypothesis^12^. Recently, investigators have detected that PVD affects the intraocular cytokine levels as measured in the aqueous, which may present another contributing factor to the role of the vitreomacular interface configuration in anti-VEGF therapy^28^. Furthermore, the presence of a PVD was associated with improved oxygenation of the retina, which may likely alters VEGF expression in the eye^29^. The finding that eyes with VMA exhibited poorer mean baseline BCVA levels (at least in CRVO) may be a sign of increased VEGF load in eyes that have not yet experienced PVD. Our study adds clinical evidence supporting a role of VMA in anti-VEGF treatment of RVO, however, further experimental studies are required to shed light onto the contributing pathomechanism.

Ongoing developments in advanced *in-vivo* imaging continue to change management paradigms in clinical practice on a frequent basis^30^. Particularly in OCT, the ever-increasing resolution and scanning speed allow to capture massive amounts of highly detailed morphologic data with each single OCT exam. Unfortunately, innovation in analysing retinal imaging data is significantly lacking behind the rapid development cycles for imaging hardware. This dissociation between hardware capabilities and limitations conferred by manual review of hundreds of images has given rise to substantial interest in automated computational analysis methods^31, 32^. Validated software algorithms have the potential to rapidly and reliably process data, capture the morphology of disease in a meaningful manner, and provide clinically relevant outcome measures to the ophthalmologist.

In our study, we use a custom-developed, fully automated segmentation algorithm to detect the posterior vitreous boundary in relation to the ILM in clinical SD-OCT data. The volumetric segmentation enables examination of the vitreomacular interface configuration as a ILM-vitreous distance map as well as three-dimensional inspection (Fig. 1), which could provide a clinically useful interpretation of the posterior vitreous, e.g. prior to vitreoretinal surgery. In addition, we employ unsupervised clustering, a data-driven, hypothesis-independent machine learning technique, to automatically group individual study eyes into clinically meaningful morphological subclasses. Our clustering approach is not limited by arbitrary thresholding or other *a-priori* constraints. Depending on the number of selected clusters, the unsupervised *k*-means classifier identified clinically distinctive classes of different degrees of VMA, as well as the non-VMA class. Regardless of the number of clusters, the non-VMA class consistently demonstrated lowest BCVA gains. However, in the cohort of patients with CRVO, eyes without VMA also showed higher baseline BCVA scores, confounding these differences in BCVA gains^6^.

This study is mainly limited in its retrospective nature. Since the vitreous segmentation algorithm is currently only available for Spectralis OCT images, only 55% of the total eligible study population could be used for analysis. Potentially, the exclusion of patients imaged with other SD-OCT devices may have caused selection bias. However, the inclusion of prospectively collected, randomized controlled trial data confers many advantages, including a standardized SD-OCT acquisition protocol, treatment regimen and BCVA measurement. Our algorithm is currently being extended to also segment images of other OCT machines. As the algorithm is based on a 2D approach, feature discontinuities across different B-scans lead to a slightly reduced smoothness in the final 3D segmentation results. A feature representation in 3D would result in a smoother segmentation, however, this benefit is mitigated by the highly anisotropic voxel size of the Spectralis instrument (~3.8 × 11.1 × 118 µm). Furthermore, the non-VMA class detected by our clustering approach may contain both patients with completely attached posterior vitreous (where the ILM-vitreous distance is *0*) and with complete PVD (where the vitreous boundary is antepositioned beyond the OCT scanning range and the distance is therefore unknown). In the age group of patients enrolled in the CRYSTAL and BRIGHTER studies, the prevalence of completely attached posterior vitreous is reported to be low (14% in patients aged 60–69 years); thus, eyes with complete PVD are likely representing the majority of the non-VMA cluster^33^. Future work will address the use of Fourier-space features to investigate alternative means of introducing a degree of rotation invariant features^34, 35^. Furthermore, deep learning approaches, for instance convolutional neural networks (CNN), have been shown as a promising way forward for image segmentation in retinal OCT data^36^.

In conclusion, we have demonstrated the feasibility of fully automated computational diagnosis of VMA in a large-scale clinical SD-OCT dataset. Data-driven unsupervised clustering of the segmentation results yielded clinically meaningful subclasses of non-VMA as well as different degrees of VMA. In macular oedema secondary to branch and central RVO, eyes with VMA afforded larger functional benefits from anti-VEGF therapy, although this association diminished after adjustment for baseline BCVA. Automated computational analysis of ophthalmic imaging data may help to characterize morphologic changes on OCT effectively, and may facilitate clinical management in a field of high-throughput patient care in the future.

### Description of the segmentation algorithm

In order to fully automatically segment the posterior vitreous boundary, we designed an image processing pipeline consisting of four major steps: (1) feature extraction, (2) 5 class region prediction, (3) 3D graph theoretic segmentation and (4) auto-context refinement.

We treat the vitreomacular segmentation problem as a voxel-wise region probability prediction problem and define the retinal layer surfaces as the boundaries between these regions. The regions used in this algorithm are from exterior to interior the vitreous, the posterior vitreous boundary, the space between the posterior vitreous boundary and the inner limiting membrane, the retina (i.e. the space between ILM and the retinal pigment epithelium (RPE)) and the space exterior of the RPE.

The final surface segmentation is then defined as the set of four boundaries (i.e. the top and the bottom surface of the posterior vitreous boundary, the ILM and the RPE) that separate the interior from the exterior part of the OCT volume so that the corresponding regions lie above respectively below them.

### Rotation invariant eigenfeatures

The performance of the machine learning algorithm used in a late stage of the pipeline depends heavily on the descriptiveness of the features used to train it. Instead of hand crafting suitable features we used the principle component analysis (PCA) of small image patches as way of learning convolution filters. To furthermore increase the descriptive power of these features, a degree of rotation invariance was introduced by computing the local orientation of small image patches centred around each voxel and rotating the corresponding convolution filter accordingly^37^.

For each image patch *I* the raw image moment is defined as ${M}_{pq}=\sum _{x}\sum _{y}{x}^{p}\ast {y}^{q}\ast I[x,y]$ and the centroid as $\bar{x}=\frac{{M}_{10}}{{M}_{00}}$ resp. $\bar{y}=\frac{{M}_{01}}{{M}_{00}}$. With this the central image, moment can be defined as ${\mu }_{pq}=\sum _{x}\sum _{y}{(x-\bar{x})}^{p}\ast {(y-\bar{y})}^{q}\ast I[x,y]$ and ${\mu }_{pq}^{^{\prime} }=\frac{{\mu }_{pq}}{{M}_{00}}$. The covariance matrix of the image patch can now be expressed as $$cov(I)=[\begin{array}{cc}{\mu }_{20}^{^{\prime} } & {\mu }_{11}^{^{\prime} }\\ {\mu }_{11}^{^{\prime} } & {\mu }_{02}^{^{\prime} }\end{array}]$$. The eigenvectors of this covariance matrix correspond to the major/minor axes of the image patch. This can be used to estimate the local orientation of the image patch by computing the angle of the largest eigenvector $\phi (I)=arc\tan (\frac{2\ast {\mu }_{11}^{^{\prime} }}{{\mu }_{20}^{^{\prime} }-{\mu }_{02}^{^{\prime} }})/2$. In order to mitigate border effects, instead of square image patches we use circular image patches. This can be achieved by replacing the raw image moment *M*_*pq*_ by the masked image moment ${\hat{M}}_{pq}=\sum _{x}\sum _{y}{x}^{p}\ast {y}^{q}\ast I[x,y]\ast mask[x,y]$ with *mask*[*x*, *y*] being 0 outside and 1 inside the circular patches.

**Figure 3:**
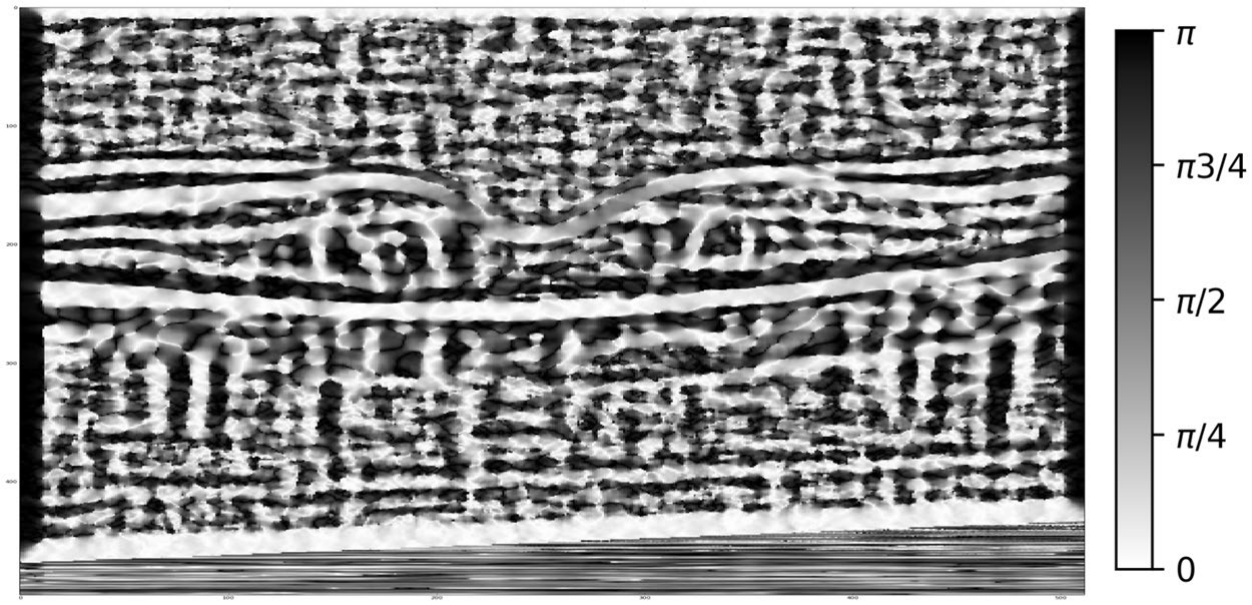
Local image orientation computed using masked local image moments.

By extracting sliding windows *I* of size 21 × 21 pixels and computing *φ*(*I*) for each window a map of the local orientations can be generated (Fig. 3).

**Figure 4:**
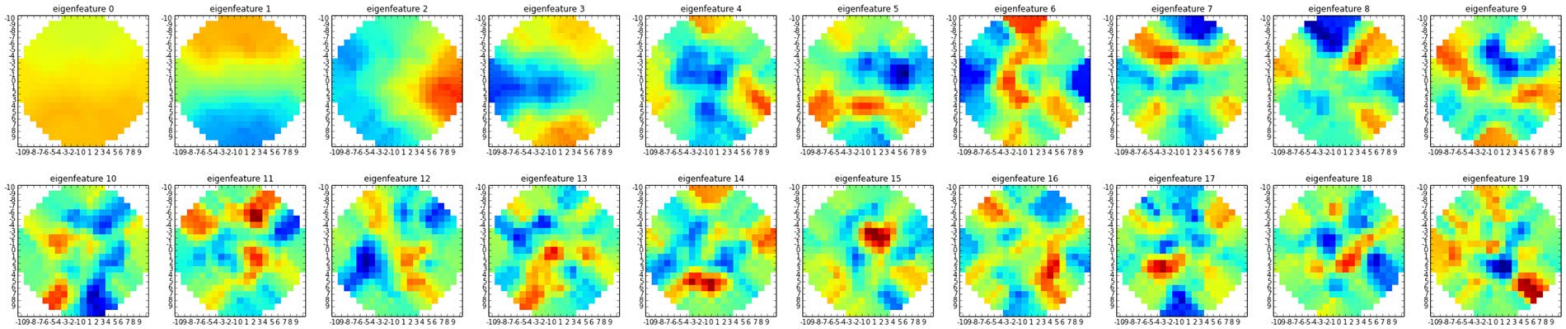
Masked, rotation invariant eigenvectors used as convolution kernels.

Using the local image orientation, we can now extract small 21 × 21 masked image patches *I* and rotate them by *φ*(*I*). This rotated image patches are the used in a principal component analysis (PCA) to compute the first 20 eigenvectors of the patches. These eigenvectors (shown in Fig. 4) are then used as convolution kernels resulting in the image features used for the machine learning algorithm, as described in detail elsewhere^20^.

### Voxelwise region prediction

After computing the voxel wise features as described above we trained a random forest classifier^38^ on 337 B-scans of 88 macula-centred SD-OCT scans in which the posterior vitreous boundary was manually annotated by trained readers of the Vienna Reading Center as described previously^39^. Since only the exterior boundary of the vitreous cortex is of interest and was thus annotated we assumed the vitreous cortex region to be at least 3 voxels thick and excluded 20 voxels above the annotation from the training.

**Figure 5:**
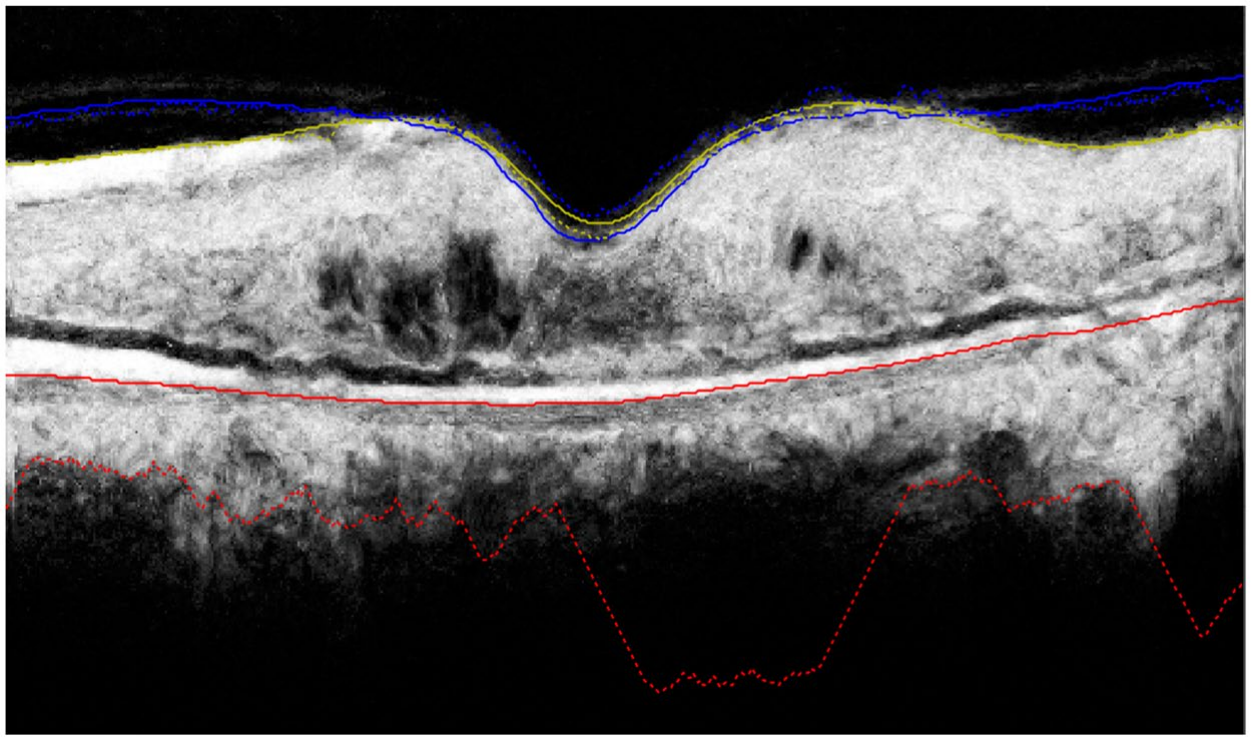
Prediction of the ILM-RPE class. Solid lines: Manual annotation (ground truth); Dashed lines: Base surface segmentation.

For each voxel in the training set, the random forest classifier is presented with the feature vector computed above and a label encoding which of the 5 classes the voxel belongs to (above vitreous, VMI, VMI-ILM, ILM-RPE and below RPE). Using this information, the classifier is able to predict the probability that a previously unseen voxel belongs to any of the 5 classes. Figure 5 illustrates an example of the probability map for the ILM-RPE class.

### Surface extraction

In order to extract the surface segmentation from the generated probability maps we used a 3D graph theoretic approach described by Garvin *et al*.^40^. This graph cut approach requires for each voxel a cost term that represents the penalty or benefit of that voxel being below or above the final segmentation. Since the result of the classifier used in the previous step directly encodes this information, the generated probability maps can directly be used as region cost terms. For example, to segment the ILM the sum of the regions vitreous, vitreous cortex and between vitreous cortex and ILM is used as region cost in the graph theoretic approach. The dashed lines shown in Fig. 5 show the result of such a segmentation.

### Iterative improvement

As shown in Fig. 5, the segmentation results (while mostly correct) contain severe segmentation errors. In order to improve the result, we apply a technique called auto-context in a similar manner as described previously^37, 41^. We compute for each voxel the axial distance to all extracted surfaces and extract raw probability samples of certain classes in the close neighbourhood of the voxels (e.g. what is the probability that the voxel above me belongs to the ILM-RPE region?). These distances and samples are then merged with the image features described previously. Together they are used to train a second classifier that can leverage the information learned by the first classifier. This process can be repeated multiple times to iteratively improve the segmentation results.

**Figure 6:**
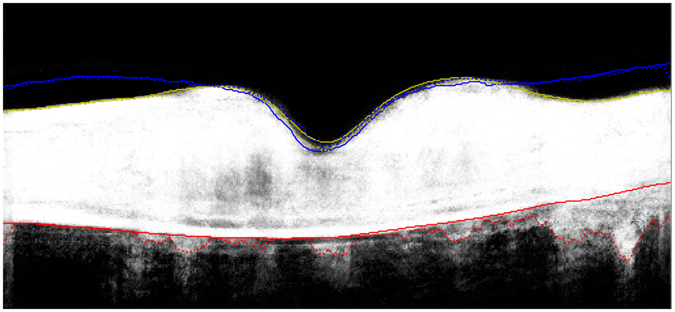
Prediction of the ILM-RPE class after two auto-context iterations. Solid lines: Manual annotation (ground truth); Dashed lines: Refined surface segmentation.

The probability map and the surface segmentation after two such iterations can be seen in Fig. 6. Note that the large segmentation errors are mostly gone and the segmentation approaches the ground truth.

### Validation of segmentation algorithm

**Figure 7:**
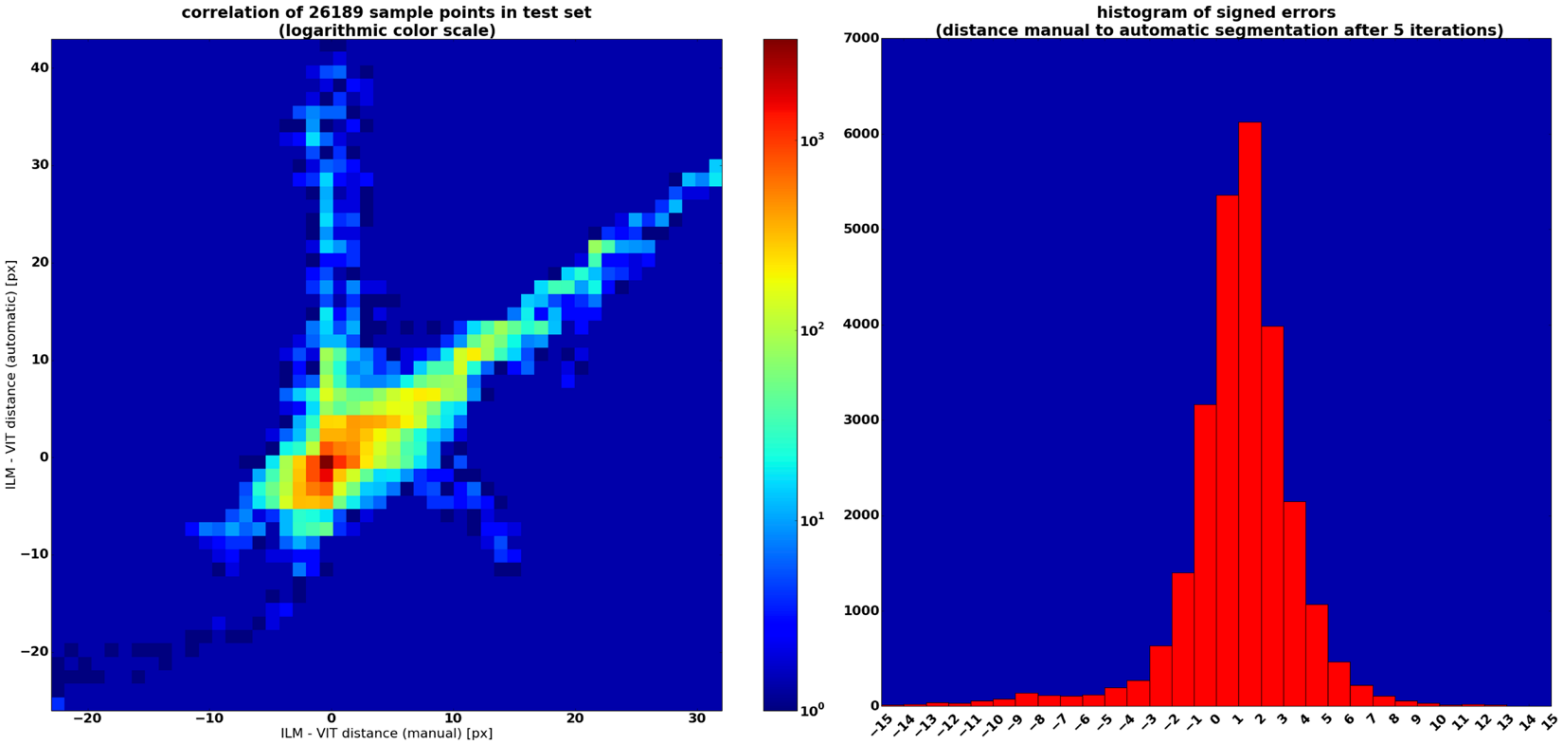
Difference between manual and automatic segmentation (in pixels). Left: correlation between manual and automatic segmentation (note logarithmic colour scale), Right: Histogram of errors.

The segmentation accuracy of the proposed method was tested by comparing the pixel distance between the ILM and the posterior vitreous boundary as determined by the algorithm against the manual annotation. For this purpose, 11 SD-OCT volumes (with a total of 26,189 annotated A-scans) were randomly chosen from the data set and excluded from the training set used in the steps described above. For each A-scan the difference between the automatic and the manual segmentation was calculated (Fig. 7).

**Figure 8:**
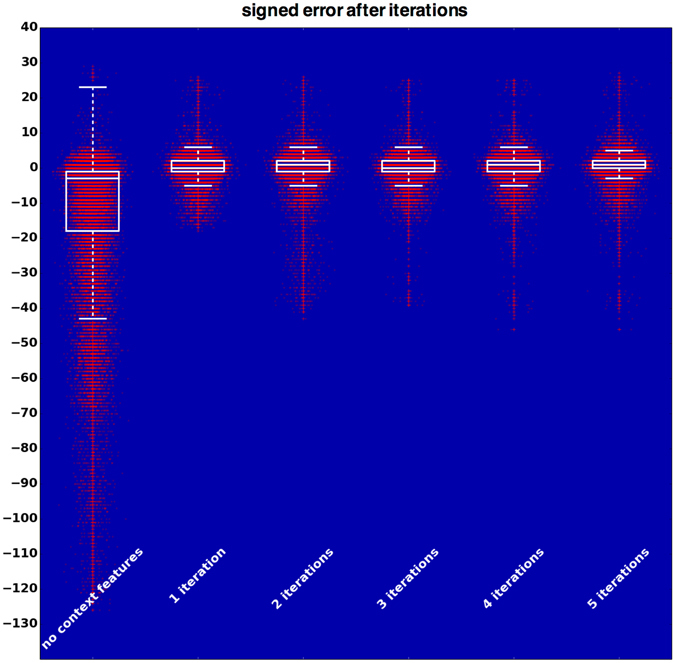
Increase in segmentation accuracy due to auto-context approach. Red dots indicate the signed error of each A-scan in the test set (with random horizontal displacement for visualization purposes), white markers show quartile and median error values.

As shown in Fig. 8, the iterative improvement method described above increases the segmentation accuracy significantly after only a few iterations. However, due to the higher computation costs and the diminishing increase in segmentation accuracy, only few iterations are practicable.
